# Automatic optic nerve head localization and cup-to-disc ratio detection using state-of-the-art deep-learning architectures

**DOI:** 10.1038/s41598-020-62022-x

**Published:** 2020-03-19

**Authors:** Keunheung Park, Jinmi Kim, Jiwoong Lee

**Affiliations:** 1 Department of Ophthalmology, Pusan National University College of Medicine, Busan, Korea; 2 Department of Biostatistics, Clinical Trial Center, Biomedical Research Institute, Pusan National University Hospital, Busan, Korea; 3 Biomedical Research Institute, Pusan National University Hospital, Busan, Korea

**Keywords:** Translational research, Computer science

## Abstract

Computer vision has greatly advanced recently. Since AlexNet was first introduced, many modified deep learning architectures have been developed and they are still evolving. However, there are few studies comparing these architectures in the field of ophthalmology. This study compared the performance of various state-of-the-art deep-learning architectures for detecting the optic nerve head and vertical cup-to-disc ratio in fundus images. Three different architectures were compared: YOLO V3, ResNet, and DenseNet. We compared various aspects of performance, which were not confined to the accuracy of detection but included, as well, the processing time, diagnostic performance, effect of the graphic processing unit (GPU), and image resolution. In general, as the input image resolution increased, the classification accuracy, localization error, and diagnostic performance all improved, but the optimal architecture differed depending on the resolution. The processing time was significantly accelerated with GPU assistance; even at the high resolution of 832 × 832, it was approximately 170 ms, which was at least 26 times slower without GPU. The choice of architecture may depend on the researcher’s purpose when balancing between speed and accuracy. This study provides a guideline to determine deep learning architecture, optimal image resolution, and the appropriate hardware.

## Introduction

Recently, there have been considerable advances in computer technology and breakthroughs in artificial-intelligence algorithms for “deep learning”. In the field of ophthalmology, automated image analysis using deep learning is attracting increasing attention, and researchers have reported that it can outperform a human doctor^1,2^. One of the significant innovations in image-classification deep-learning algorithms was AlexNet^3^, which was developed by Alex Krizhevsky. AlexNet competed in the ImageNet Large Scale Visual Recognition Challenge (ILSVRC) in 2012 and was overwhelmingly victorious. This algorithm opened a new horizon of computer vision using deep convolutional neural networks (CNNs).

Since AlexNet was developed, there have been numerous modifications to CNN architectures. One of these modifications—and another significant leap—was ResNet^4^, which is famous for its “residual connection.” Before ResNet, deep CNN architectures had a “degradation problem”: with the increase of the depth of a neural network, the accuracy becomes saturated and decreases rapidly^5,6^. ResNet solved this problem via residual connection, which adds the output of the lower layer to the higher layer. This direct addition of the lower-layer output delivers undegraded information to the higher layer and makes deep neural network training more effective^4^. ResNet won 1^st^ place in the 2015 ILSVRC.

Another state-of-the-art approach for solving the degradation problem is DenseNet, which was introduced by Huang *et al*.^7^ in 2016. This architecture feed-forwards each layer to every other layer. ResNet also forwards the output of the lower layer directly to the higher layer; however, in contrast, DenseNet forwards the lower layer to all the other layers and does not combine features using additive summation but concatenates them. Via the feed-forwarding of the lower-layer outputs, it can keep the feature maps unchanged, and the last layer can make a decision based on all of the feature maps.

Recently proposed computer-vision algorithms are not limited to “classifying” images. They can detect the location of an object in an image (object detection) and even separate the exact contours of objects (object segmentation). The ResNet and DenseNet algorithms have been employed for object detection, exhibiting good performance^8,9^. Another new object-detection algorithm is YOLO-V3^10^, which has a “very” high speed of detection and accuracy comparable to that of other state-of-the-art architectures. In a previous study, YOLO V3 detected an object in an average of 51 ms, indicating that it can process images in a video in real time. This detection speed is three times higher than that of the Single Shot MultiBox Detector (SSD)^11^, which is famous for its fast detection.

Researchers who plan to develop an automatic fundus image reading system using a deep-learning algorithm face the following common questions. Which deep-learning architecture is the most suitable for ophthalmology? How high of an input image resolution is required? How much is the speed of the system improved by using a graphics processing unit (GPU)? If a GPU cannot be used, what is the resulting performance degradation? How exactly does the deep-learning algorithm locate the lesions of interest?

The objective of this study was to answer the foregoing questions. We compared the state-of-the-art deep-learning architectures YOLO V3, ResNet, and DenseNet with regard to their processing time, localization accuracy, and classification accuracy. The target object was the optic nerve head (ONH), which is the most prominent feature in fundus images, and the performance for classifying its vertical cup-to-disc ratio (VCDR), which is a widely accepted index for the assessment of glaucoma diagnosis, was evaluated^12^. We also evaluated the effects of various input image resolutions on the performance to determine the optimal image size for fundus photograph analysis.

## Methods

This retrospective study was performed in accordance with the tenets of the Declaration of Helsinki. The study was approved by the Institutional Review Board (IRB) of Pusan National University Hospital; the requirement for patient consent was waived by the IRB because of the retrospective nature of the study.

All training and test data were for subjects who had visited the glaucoma clinic at Pusan National University Hospital (South Korea) from 2010 to 2018. The demographic characteristics of the training group are presented in Table 1. The training dataset consisted of 1,959 eyes of 1,068 subjects and was not labeled by diagnosis. Therefore, normal fundi, as well as data for subjects with glaucoma and other optic neuropathies, were included. However, eyes with retinal disease or severe media opacity (such as cataracts) were excluded. The mean age was 58.2 ± 16.1 (mean ± standard deviation (SD)), and the female/male ratio was 531/537. A total of 1,959 records from the training dataset were randomly split into training data and validation data at a ratio of 9:1. The validation data were used to check the fitness of the neural network during training to prevent overfitting.

**Table 1:** Demographic characteristics of the training group.

	Values
Total number of patients (female/male)	1,068 (531/537)
Total number of eyes	1959
Age (mean ± SD)	58.2 ± 16.1
Number of eyes binned by VCDR	
VCDR < 0.4	111 (5.7%)
0.4 ≤ VCDR < 0.5	125 (6.4%)
0.5 ≤ VCDR < 0.6	279 (14.2%)
0.6 ≤ VCDR < 0.7	467 (23.8%)
0.7 ≤ VCDR < 0.8	516 (26.3%)
0.8 ≤ VCDR < 0.9	379 (19.3%)
0.9 ≤ VCDR	82 (4.2%)

SD: standard deviation, VCDR: vertical cup-to-disc ratio

Another dataset, which contained 204 eyes of 204 subjects, was used as the test dataset. There was no patient overlap between the training and test datasets. For all the subjects in the test group, a retrospective review of the detailed results of ophthalmic examinations was performed. These ophthalmic examinations included the following measurements: Goldmann applanation tonometry, slit-lamp examination, funduscopy, biometry using IOLMaster (Carl Zeiss Meditec, Dublin, CA, USA), Humphrey visual-field tests (Carl Zeiss Meditec, Dublin, CA, USA), central corneal thickness (CCT) evaluation using ultrasonic pachymetry (Pachmate; DGH Technology, Exton, PA, USA), keratometry using the Auto Kerato-Refractometer (ARK-510A; NIDEK, Hiroshi, Japan), and Cirrus (Carl Zeiss Meditec, Dublin, CA, USA) high-definition optical coherence tomography (HD-OCT). Glaucomatous optic neuropathy was defined by one or more of the following criteria being satisfied: focal or diffuse neuroretinal rim thinning, localized notching, cup-to-disc ratio asymmetry (≥0.2), and the presence of retinal nerve fiber layer defects congruent with visual-field defects^13^. Normal subjects were defined as those with no history of ocular disease, an intraocular pressure (IOP) of <21 mmHg, the absence of glaucomatous optic disc appearance, and a normal visual field.

### Deep-learning architectures and training

We trained the state-of-the-art object-detection architectures YOLO V3^10^, ResNet50, and DenseNet201. The script codes for these architectures and darknet C source codes were directly downloaded from the homepage of the darknet^14^ and compiled in a Windows console application using Microsoft Visual Studio 2015. The hardware used included an Intel 8^th^ generation central processing unit (CPU) (i5–8400, 2.81 GHz, 32 GB main memory) and an NVIDIA Titan Xp (12 GB; Santa Clara, CA, USA). For testing the architectures without a GPU (i.e., only-CPU mode), the NVIDIA Titan Xp was physically detached from the computer.

Deep learning architectures were trained to find the location of the optic nerve head (ONH) and determine its vertical cup-to-disc ratio (VCDR). This is because the ONH is one of the most prominent anatomical structures in fundus images, so we can easily draw the bounding box containing the ONH, while minimizing human error when preparing training and test data. This means more objective comparisons of deep learning architectures are possible. VCDR of the ONH is widely accepted as one of the important indicators of glaucoma^15^. By detecting VCDR of the ONH, we can also compare the glaucoma diagnostic performance of deep learning architectures. VCDR is a continuous number from 0 to 1 and, to label it, the number was binned by 0.1 starting from 0.4. A VCDR < 0.4 was labeled as ‘0.4’ (which actually meant ≤0.4) because the minimum cutoff value of the abnormal VCDR was reported as 0.5^15^ and physicians also had greater disagreement when determining a VCDR < 0.4^16^.

We developed a custom annotation software to mark various retinal lesions and ONH. Using this software, the ONH was manually marked, and the VCDR was automatically determined according to the ONH parameters exported from Cirrus HD-OCT. To accelerate the training, we used a pretrained neural network weight file for the COCO dataset^17^ and fine-tuned the model. The COCO dataset contains approximately 330,000 images in 80 object categories. A network pretrained on a large and diverse dataset such as COCO captures universal features, e.g., curves and edges, that are relevant and useful for most classification problems. In a previous study, the fine-tuning method for medical images outperformed or—in the worst case—performed as well as a model trained from scratch^18^. We performed a total of approximately 30,000 iterations, with a batch size of 64. The detailed training time and number of iterations are summarized in Supplementary Table S1. We monitored the loss of the validation set to evaluate the performance for unpresented data and stopped the training when no improvement of the loss was observed. The same method was applied for all the architectures (YOLO V3, ResNet50, and DenseNet201).

**Figure 1:**
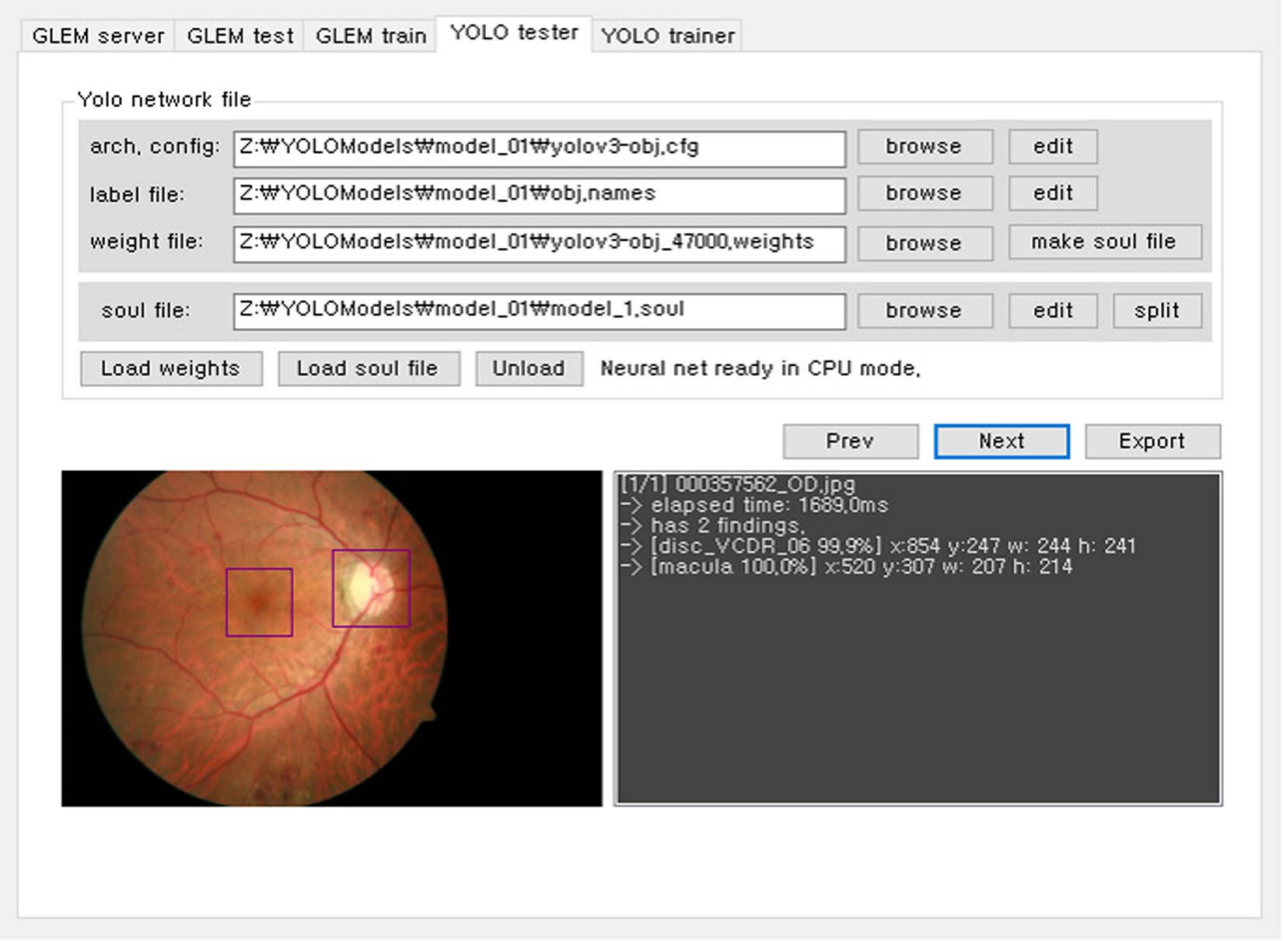
Custom-developed deep-learning architecture tester software.

To test the architectures, we developed a custom software for Windows using Microsoft Visual Studio 2015 (Fig. 1). This software employed the original C code downloaded from the homepage of the darknet^14^ and transformed it into the Windows form application using the common language runtime and marshaling functions in Visual Studio 2015. All of the test images were automatically supplied to the software one-by-one (i.e., a batch process was not used), and the detection time, coordinates of the detected object, and predicted VCDR were calculated and exported automatically. The predicted location of the object (the ONH) was compared with the ground truth location of the object. Using the formulae given in the section on statistical analyses, we calculated the intersection over union (IoU) and mean average precision (mAP) to examine the accuracy of the localisation. The predicted VCDR was compared with the ground truth VCDR, and the mean absolute (MAE) error was determined to evaluate the overall classification performance of the deep-learning architecture. The MAE was binned by 0.1 to evaluate its distribution.

### Spectral-domain optical coherence tomography (SD-OCT) imaging

The Cirrus SD-OCT instrument (Carl Zeiss Meditec, Dublin, CA, USA) was used to measure the ONH parameters. After pupil dilation using 0.5% tropicamide and 0.5% phenylephrine, a single scan of each eye was performed using the Optic Disc Cube 200 × 200 protocol. After the subject was seated and properly aligned, the eye was brought into view using the mouse-driven alignment system, and the line-scanning image was focused by adjusting for refractive error. The ONH was then shown at the center of the live image, and further centering (Z-offset) and enhancement were performed. A laser was scanned over a 6 × 6-mm^2^ area, yielding a cube of data consisting of 200 × 200 A-scans to make B-scans (40,000 points) in approximately 1.5 s (27,000 A-scans/s). For quality control, only high-quality scans—defined by a minimum signal strength of 6; the absence of retinal nerve fiber layer (RNFL) discontinuity, misalignment, and segmentation failure; and the absence of involuntary movement of the eye or blinking artifacts—were used for analysis. The VCDR (length ratio of the vertical line through the cup center to the same vertical line extending to the disc margin) was automatically calculated via a Carl Zeiss Meditec ONH analysis algorithm (software version 5.0) implemented in a Cirrus HD-OCT. The algorithm identified Bruch’s membrane opening as the optic disc margin and quantified the neuroretinal rim via advanced minimum-cross section calculation.

### Statistical analyses

To evaluate the localization accuracy, the intersection over union (IoU) and mean average precision (mAP) were used. These metrics are very popular and are often used in object-detection challenges such as the PASCAL VOC challenge^19^. The IoU is defined as the area of intersection divided by the area of union and indicates the similarity between the predicted bounding box and the ground-truth bounding box. The formula is as follows:

1 $$\begin{array}{c}IoU=\frac{Area\,of\,overlap\,between\,A\,and\,B}{Area\,of\,union\,A\,and\,B}\\ A=predicted\,bounding\,box,\,B=Ground\,truth\,bounding\,box.\end{array}$$

mAP is a default metric of precision in the PASCAL VOC competition and also indicates the accuracy of object-detection algorithms. It is the percentage of true positive detections among all positive detections, as follows:

2 $$mAP=\frac{True\,positive}{True\,positive+False\,positive}$$

Among all the detected objects (true positive + false positive detection), a true positive is defined by the IoU of the detected object exceeding a threshold. Depending on the IoU threshold, various mAPs may be defined; among them, mAP_50_ corresponds to a threshold of 50%.

To evaluate the classification accuracy, the mean absolute error (MAE) between the predicted VCDR and the ground-truth VCDR was used. We also calculated another metric called N_AE_, which represents the number of subjects whose absolute error (AE) of the predicted VCDR satisfied a certain limitation. For example, N_AE <0.1_ represents the number of subjects whose AE of the predicted VCDR was <0.1.

The Shapiro–Wilk test was performed to evaluate the normality of the continuous data. To compare the parameters between normal subjects and glaucoma patients, we used the Student’s t-test or the Mann–Whitney U test, depending on the normality of the data. The chi-square test was used for categorical variables, and generalized estimating equations (GEEs) were used for binary data, with the logit link function and an unstructured correlation matrix. The Friedman test was performed to compare the performance of the deep-learning architectures, and Wilcoxon’s signed-rank test was performed for post-hoc analysis. To compare the diagnostic performance, we used the area under the receiver operating characteristic curve (AUROC). To calculate the difference between two AUROCs, DeLong’s method^20^ was used. For statistical analyses, SPSS (version 21.0 for Windows; Chicago, IL, USA) and MedCalc (version 12.5 for Windows; Ostend, Belgium) were used, and *P* < 0.05 (single comparison) and *P* < 0.017 (multiple comparison) were considered to indicate statistical significance.

## Results

A total of 204 eyes from 204 subjects (95 healthy normal subjects and 109 glaucoma patients) were employed for the performance test group. The demographic characteristics of the subjects are presented in Table 2. There were significant differences in the age and CCT (*P* = 0.003 and 0.045, respectively), but the gender, refractive error, IOP, and axial length did not differ significantly. All the visual-field parameters were significantly different. The average visual-field mean deviation (MD) was −1.48 dB for normal subjects and −11.25 dB for glaucoma patients.

**Table 2:** Demographic characteristics of the test group.

	Normal (n = 95)	Glaucoma (n = 109)	*P* value
Age (year)	51.8 ± 22.1	60.1 ± 16.0	0.003^a^
Female/male (number)	47/48	52/57	0.801^b^
Spherical equivalence (diopter)	−1.92 ± 3.42	−1.85 ± 3.20	0.878^a^
Intraocular pressure (mmHg)	15.7 ± 3.3	15.3 ± 4.3	0.466^a^
Axial length (mm)	24.89 ± 3.14	24.60 ± 1.50	0.607^a^
Central corneal thickness (µm)	551.9 ± 46.5	539.0 ± 39.7	0.045^a^
**Visual field test**			
- Mean deviation (dB)	−1.48 ± 2.02	−11.25 ± 9.41	<0.001^c^
- Pattern standard deviation (dB)	1.84 ± 0.94	5.76 ± 3.85	<0.001^c^
- Visual Field Index (%)	98.0 ± 3.2	69.3 ± 32.3	<0.001^c^

^a^Student’s t-test.

^b^χ2 test.

^c^Mann–Whitney U test.

Values are presented as mean ± standard deviation.

The mean detection times are presented in Table 3. Without the GPU, among the three architectures, YOLO V3 was the slowest and ResNet was the fastest for all input image resolutions. For the resolution of 224 × 224, the mean detection times were 531, 314, and 394 ms for YOLO V3, ResNet, and DenseNet, respectively. When the input image resolution increased, the detection time increased roughly in proportion to the input data size. For example, the images with a 416 × 416 resolution had approximately four times more data than those with a 224 × 224 resolution and increased the time required by approximately four times. However, when the GPU was used, the detection times were significantly improved and were no longer proportional to the input resolution. They depended more strongly on the architecture, and DenseNet was the slowest in most cases. With GPU assistance, the speed of YOLO V3 was significantly improved, and the difference between the detection times of YOLO V3 and ResNet became ≤6 ms. For a resolution of 832 × 832, the detection time with GPU assistance was 171, 165, and 167 ms for YOLO V3, ResNet, and DenseNet, respectively). At lower resolutions, the mean detection times of YOLO V3 and ResNet were approximately 110 ms, while that of DenseNet was 160 ms /136 ms (224 × 224/416 × 416, respectively). The speed of DenseNet was not improved with GPU assistance at the lower resolutions.

**Table 3:** Comparison of the mean detection time for different hardware.

Resolution	Hardware	YOLO V3 (ms)	ResNet (ms)	DenseNet (ms)	*P*_*all*_^*c*^	Post-hoc analysis		
						*P*_*YR*_^*d*^	*P*_*RD*_^*e*^	*P*_*YD*_^*f*^
224 × 224	CPU only^a^	531 ± 80	314 ± 53	394 ± 66	<0.001	<0.001	<0.001	<0.001
	with GPU^b^	112 ± 32	116 ± 39	160 ± 57	<0.001	0.582	<0.001	<0.001
416 × 416	CPU only^a^	1,977 ± 228	942 ± 158	1,198 ± 189	<0.001	<0.001	<0.001	<0.001
	with GPU^b^	117 ± 27	111 ± 28	136 ± 32	<0.001	<0.001	<0.001	<0.001
832 × 832	CPU only^a^	11,365 ± 967	4,395 ± 395	6,055 ± 591	<0.001	<0.001	<0.001	<0.001
	with GPU^b^	171 ± 35	165 ± 39	167 ± 30	<0.001	<0.001	<0.001	<0.001

^a^Intel i5–8400 2.81 GHz + 32 GB RAM.

^b^NVIDIA Titan Xp 12 GB RAM.

^c^*P* value among all three architectures (Friedman test).

^d^*P* value between Yolo V3 and ResNet (Friedman test).

^e^*P* value between ResNet and DenseNet (Friedman test).

^f^*P* value between Yolo V3 and DenseNet (Friedman test).

Values are presented as mean ± standard deviation.

The localization accuracy of the detected ONH is presented in Table 4. As the input resolution increased, the accuracy of YOLO V3 and ResNet was significantly improved, but that of DenseNet—which exhibited the best accuracy at the 224 × 224 resolution—was not. At the 224 × 224 resolution, the best mean IoU was achieved by DenseNet (80.0%), followed by ResNet (69.6%) and YOLO V3 (67.7%). The best mAP_50_ was also achieved by DenseNet (99.51%), followed by ResNet (94.61%) and YOLO V3 (93.14%). As the resolution increased to 416 × 416, the localization accuracy of YOLO V3 (IoU = 79.4%, mAP_50_ = 100.00%) was significantly improved, surpassing that of ResNet (IoU = 69.0%, mAP_50_ = 95.10%); however, the best accuracy was still achieved by DenseNet (IoU = 81.2%, mAP_50_ = 100.00%). At the 832 × 832 resolution, the best localization accuracy was achieved by YOLO V3 (IoU = 81.5%, mAP_50_ = 99.02%), followed by DenseNet (IoU = 80.7%, mAP_50_ = 100.00%) and ResNet (IoU = 77.2%, mAP_50_ = 95.59%). However, the difference in the IoU between YOLO V3 (81.5%) and DenseNet (80.7%) was not statistically significant *(P* = 0.651 in Wilcoxon’s signed-rank test).

**Table 4:** Localization accuracy.

Resolution	Statistics	YOLO V3	ResNet	DenseNet	*P*_*all*_^*a*^	Post-hoc analysis		
						*P*_*YR*_^*b*^	*P*_*RD*_^*c*^	*P*_*YD*_^*d*^
224 × 224	Mean IoU, %	67.7 ± 11.3	69.6 ± 10.7	80.0 ± 9.4	<0.001^e^	0.005^f^	<0.001^f^	<0.001^f^
	mAP_50_, %	93.14	94.61	99.51	0.016^g^	0.512^g^	0.003^g^	<0.001^g^
416 × 416	Mean IoU, %	79.4 ± 6.0	69.0 ± 10.2	81.2 ± 7.2	<0.001^e^	<0.001^f^	<0.001^f^	<0.001^f^
	mAP_50_, %	100.00	95.10	100.00	<0.001^g^	0.001^g^	0.001^g^	NE
832 × 832	Mean IoU, %	81.5 ± 7.1	77.2 ± 11.2	80.7 ± 7.8	<0.001^e^	<0.001^f^	<0.001^f^	0.651^f^
	mAP_50_, %	99.02	95.59	100.00	0.053^g^	0.033^g^	0.002^g^	0.155^g^

^a^*P* value among all three architectures.

^b^*P* value between Yolo V3 and ResNet.

^c^*P* value between ResNet and DenseNet.

^d^*P* value between Yolo V3 and DenseNet.

^e^Friedman test.

^f^Wilcoxon’s signed-rank test.

^g^Generalized estimating equations (GEEs).

IoU: intersection over union, mAP_50_: mean average precision, NE: not estimable.

Values are presented as mean ± standard deviation.

The accuracy of the detected VCDR is presented in Table 5. As the input resolution increased, the classification error (MAE) generally decreased, indicating that the classification accuracy was improved. At the 224 × 224 resolution, the minimum MAE was achieved by ResNet (0.062), followed by DenseNet (0.065), but the difference was not statistically significant (*P* = 0.245 in Friedman test). YOLO V3 had the worst MAE (0.069), which was significantly different from those of ResNet and DenseNet (*P* < 0.001 and *P* = 0.001, respectively, in Friedman test). At the 416 × 416 resolution, the MAE was slightly improved to 0.062, 0.061, and 0.063 for YOLO V3, ResNet, and DenseNet, respectively, and the difference among the architectures was not significant (*P* > 0.803 in Friedman test). At the 832 × 832 resolution, the MAE was further improved to 0.053, 0.062, and 0.048, respectively, and the difference between ResNet and DenseNet was significant (*P* < 0.001 in Friedman test). N_AE <0.1_—the number of patients whose AE of the predicted VCDR was <0.1—increased as the input resolution increased. For YOLO V3, ResNet, and DenseNet, N_AE <0.1_ was 68.9%, 71.4%, and 70.9%, respectively, at the 224 × 224 resolution; 72.8%, 70.4%, and 72.8%, respectively, at the 416 × 416 resolution; and 77.2%, 73.8%, and 82.5%, respectively, at the 832 × 832 resolution. N_AE <0.2_—the number of patients whose AE of the predicted VCDR was <0.2—was 88.8%, 90.3%, and 88.8%, respectively, at the 224 × 224 resolution; 93.7%, 90.8%, and 91.7%, respectively, at the 416 × 416 resolution; and 96.6%, 93.7%, and 96.6%, respectively, at the 832 × 832 resolution.

**Table 5:** Vertical cup-to-disc ratio (VCDR) classification accuracy.

Statistics	YOLO V3	ResNet	DenseNet	*P*_*all*_^*c*^	Post-hoc analysis		
					*P*_*YR*_^*d*^	*P*_*RD*_^*e*^	*P*_*YD*_^*f*^
**Resolution 224 × 224**							
MAE^a^	0.069 ± 0.071	0.062 ± 0.083	0.065 ± 0.083	0.001^g^	<0.001^g^	0.245^g^	0.001^g^
N_AE <0.1_^b^	142 (68.9%)	147 (71.4%)	146 (70.9%)	0.156^h^	0.084^h^	0.891^h^	0.114^h^
N_AE <0.2_^b^	183 (88.8%)	186 (90.3%)	183 (88.8%)	0.126^h^	0.064^h^	0.564^h^	0.131^h^
**Resolution 416 × 416**							
MAE^a^	0.062 ± 0.062	0.061 ± 0.072	0.063 ± 0.073	0.531^g^	0.851^g^	0.820^g^	0.803^g^
N_AE <0.1_^b^	150 (72.8%)	145 (70.4%)	150 (72.8%)	0.682^h^	0.465^h^	0.465^h^	1.000^h^
N_AE <0.2_^b^	193 (93.7%)	187 (90.8%)	189 (91.7%)	0.369^h^	0.159^h^	0.655^h^	0.347^h^
**Resolution 832 × 832**							
MAE^a^	0.053 ± 0.059	0.062 ± 0.066	0.048 ± 0.063	0.047^g^	0.118^g^	<0.001^g^	0.025^g^
N_AE <0.1_^b^	159 (77.2%)	152 (73.8%)	170 (82.5%)	0.016^h^	0.376^h^	0.004^h^	0.062^h^
N_AE <0.2_^b^	199 (96.6%)	193 (93.7%)	199 (96.6%)	0.123^h^	0.099^h^	0.063^h^	0.655^h^

^a^Mean absolute error (MAE) between the detected VCDR and ground truth VCDR (mean ± standard deviation).

^b^The number (percent) of patients whose absolute error (AE) between detected VCDR and ground truth VCDR is less than 0.1 or 0.2.

^c^*P* value among all three architectures.

^d^*P* value between Yolo V3 and ResNet.

^e^*P* value between ResNet and DenseNet.

^f^*P* value between Yolo V3 and DenseNet.

^g^Friedman test.

^h^Generalized estimating equations (GEEs).

Values are presented as mean ± standard deviation.

**Figure 2:**
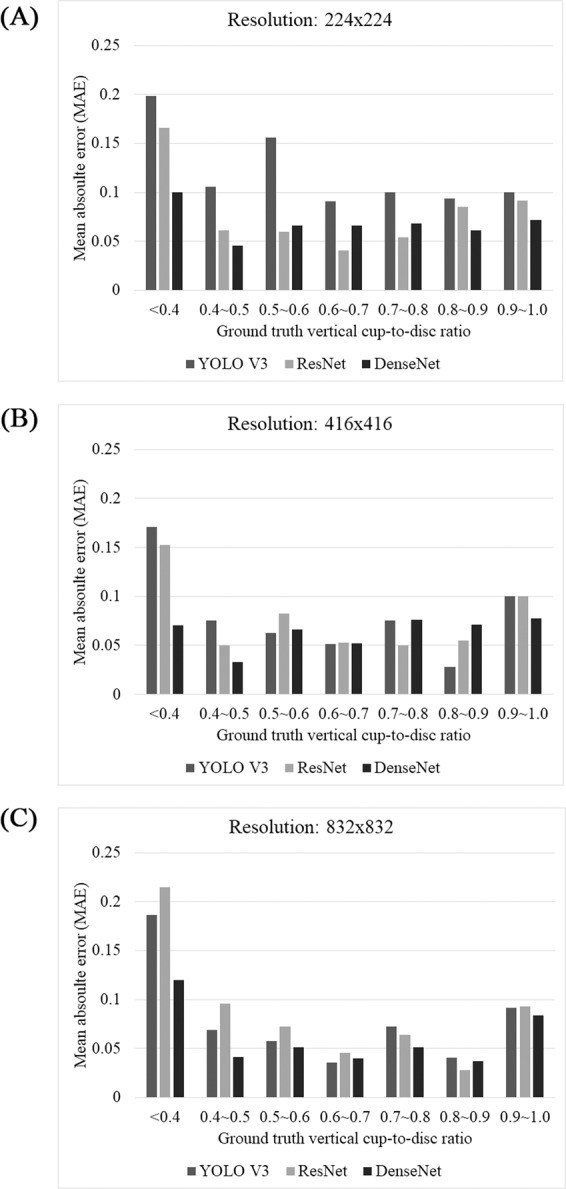
Vertical cup-to-disc ratio (VCDR) classification accuracy. Overall mean absolute error (MAE) tended to be high for patients (ground truth) with VCDR < 0.4 or VCDR > 0.9 and almost all other MAEs were <0.1. That is, excluding the patients with an extremely low or high VCDR, the MAE for the prediction of the VCDR was almost always <0.1.

The distribution of the classification error (MAE) binned by the ground-truth VCDR is presented in Fig. 2. Generally, the MAE tended to be high for patients (ground truth) with VCDR < 0.4 or VCDR > 0.9. With the exception of these two VCDR ranges, almost all other MAEs were <0.1. That is, excluding the patients with an extremely low or high VCDR, the MAE for the prediction of the VCDR was almost always <0.1.

**Figure 3:**
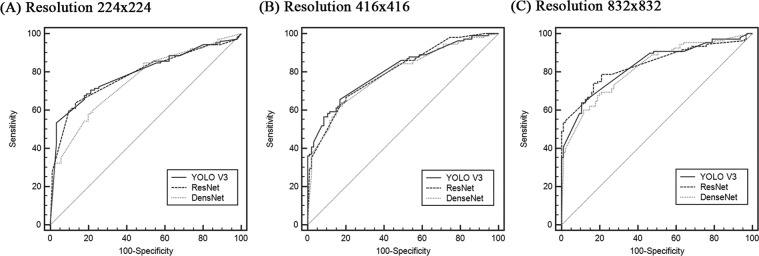
Diagnostic performance of the detected vertical cup-to-disc ratio (VCDR).

The glaucoma diagnostic performance of the detected VCDR is presented in Table 6 and Fig. 3. The overall diagnostic performance improved with the increase of the input resolution. At the 224 × 224 resolution, the best AUROC was achieved by YOLO V3 (0.793), followed by ResNet (0.785) and DenseNet (0.754). At the 416 × 416 resolution, the best AUROC was achieved by YOLO V3 (0.810), followed by ResNet (0.799) and DenseNet (0.787). At the 832 × 832 resolution, ResNet (0.838) slightly surpassed YOLO V3 (0.832), and DenseNet was still the worst (0.818). However, the performance did not differ significantly among the architectures (*P* > 0.129 for all resolutions).

**Table 6:** Diagnostic performances of detected vertical cup-to-disc ratio (VCDR).

	AUROC (CI)	*P*_*YR*_^*a*^	*P*_*RD*_^*b*^	*P*_*YD*_^*c*^
**Resolution 224 × 224**				
- YOLO V3	0.793 (0.729–0.848)	0.799	0.240	0.129
- ResNet	0.785 (0.721–0.841)			
- DenseNet	0.754 (0.687–0.813)			
**Resolution 416 × 416**				
- YOLO V3	0.810 (0.749–0.861)	0.602	0.574	0.278
- ResNet	0.799 (0.737–0.852)			
- DenseNet	0.787 (0.724–0.841)			
**Resolution 832 × 832**				
- YOLO V3	0.832 (0.773–0.881)	0.768	0.257	0.435
- ResNet	0.838 (0.780–0.886)			
- DenseNet	0.818 (0.758–0.869)			

^a^*P* value between YOLO V3 and ResNet (DeLong’s method).

^b^*P* value between ResNet and DenseNet (DeLong’s method).

^c^*P* value between YOLO V3 and DenseNet (DeLong’s method).

Values are presented as mean ± standard deviation.

AUROC: area under receiver operating characteristics, C.I.: confidence interval.

## Discussion

The main objectives of this study were to compare the performance of the state-of-the-art deep-learning architectures for object detection: YOLO V3, ResNet, and DenseNet. We evaluated the performance (mean detection time, localization accuracy, classification accuracy, and diagnostic power) with different input image resolutions. The overall accuracy improved as the input resolution increased. At a low resolution of 224 × 224, the mean detection time was approximately ≤0.5 s for all architectures without GPU assistance. DenseNet exhibited a high localization accuracy at the low resolution (IoU 80.0%, mAP_50_ 99.51%). The VCDR classification accuracy (MAE) at the low resolution was 0.069, 0.062, and 0.065 for YOLO V3, ResNet, and DenseNet, respectively; the difference was not significant. At a high resolution of 832 × 832, the mean detection time without GPU assistance increased roughly in proportion to the input data size (eight times more data than the case of 224 × 224). It was 11,365, 4,395, and 6,055 ms for YOLO V3, ResNet, and DenseNet, respectively, without GPU assistance. With GPU assistance, the detection time was slightly increased (compared with the low-resolution case) to approximately 170 ms for all architectures, but the detection was still significantly faster than that with the CPU only. The localization accuracy (IoU) was improved to 81.5%, 77.2%, and 80.7% for YOLO V3, ResNet, and DenseNet, respectively. YOLO V3 became slightly better than DenseNet, but the difference was not significant. Additionally, the classification accuracy of the VCDR (MAE) improved to 0.053, 0.062, and 0.048, respectively. In the field of ophthalmology, this study is the first to compare the performance of state-of-the-art object-detection architectures.

There have been two major studies to date: one^21,22^ involved object detection of the ONH in an entire fundus photograph, and the other^2,23,24^ involved classification of the ONH in a cropped image. Object detection involves not only classifying every object in an image but also localizing them by drawing the appropriate bounding box. This makes object detection far more difficult than image classification. In most ONH classification studies, the ONH had a higher resolution in the cropped fundus photograph than in the whole fundus image supplied to the object-detection algorithm. Our results indicate that the overall performances were all improved with the increase of the input resolution. Because object-detection algorithms are in a more difficult condition, their classification performance is unlikely to be better than that of classifier algorithms.

Object detection of the ONH has not been intensively studied. Alghamdi *et al*.^21^ reported a combined architecture for localization and classification of the ONH. Their model used a cascade classifier to locate the ONH and then used a CNN to classify the ONH into one of three categories: normal, suspicious, or abnormal. They tested model on different public datasets and reported a localization accuracy of 86.71–100.0%. However, although the cascade classifier performed well for high-quality images, the performance was significantly degraded for lower-quality images. Tan *et al*.^22^ reported segmentation of the ONH, fovea, and retinal vasculature using a single CNN. The localization accuracy (IoU) of segmenting the ONH was 0.6210. However, this algorithm took 3750.55 s (approximately 1 h) on average to completely segment a fundus image, and classification was not performed.

ONH classification algorithms have been studied more frequently than object-detection algorithms. Lim *et al*.^23^ evaluated the performance of a CNN architecture for VCDR classification. The MAE of the detected VCDR was 0.2302. Recently, efforts have been directed toward the detection (classification) of glaucoma using deep-learning algorithms. In 2015, Chen *et al*.^24^ developed a CNN architecture called ALADDIN (glaucoma detection based on deep learning) and reported that its diagnostic performance (AUROC) was 0.838 (ORIGA, online retinal fundus images for glaucoma analysis) and 0.898 (SECS datasets). More recently, in 2018, Christopher *et al*.^2^ evaluated the performance of three deep-learning architectures—VGG16, Inception V3, and ResNet50—for glaucoma detection. They trained the neural networks with a large number of fundus images (14,822) and reported an AUROC of 0.91 for distinguishing glaucomatous optic neuropathy from healthy eyes. This accuracy is almost as good as that of a glaucoma expert. In their study, ResNet was significantly better than VGG16 and Inception V3.

In this study, the best AUROC was achieved by ResNet (0.838, which is the same as that of the ALADDIN architecture and lower than that reported by Christopher *et al*.^2^). However, in contrast to previous studies, our diagnostic performance results were based on only the VCDR. The VCDR is not the only consideration of ophthalmologists for identifying abnormalities in the ONH. ONH images contain additional information related to glaucoma diagnosis, such as lamina dot signs, bayonetting of vasculatures, RNFL defects, disc hemorrhages, tilting of the ONH, and peripapillary atrophy. Because the objective of our study was not evaluating glaucoma detection but evaluating the performance of object detection and classification represented by the VCDR, the glaucoma diagnostic performance could have been underestimated. However, even though only the VCDR was used for glaucoma diagnosis, the performance was comparable to that achieved in previous studies.

To train the neural networks with an objective VCDR value, we used the ONH parameter measured via Cirrus HD-OCT. Because the ONH area can vary up to fivefold, there is no VCDR that defines absolute pathological cupping^25^. However, Mwanza *et al*.^26^ studied the glaucoma diagnostic performance of Cirrus HD-OCT and reported that the VCDR exhibited good diagnostic performance (AUROC = 0.951). Diagnostic performance of the VCDR was not significantly different from the average RNFL thickness (AUROC = 0.950) and the RNFL thickness at clock-hour 7 (AUROC = 0.957). Moreover, in another study, the ONH parameters of Cirrus HD-OCT for glaucomatous eyes showed excellent reproducibility. In particular, the intravisit intraclass correlation coefficient (ICC) of the VCDR was 97.7%, and the intervisit ICC was 97.2%^27^. In another study^28^, the ICC of the VCDR for normal eyes was >98.0%. Resch *et al*.^29^ compared Cirrus HD-OCT with confocal scanning laser ophthalmoscopy (Heidelberg retinal tomography, HRT 3). They reported a strong correlation of the VCDR between the two modalities (R^2^ = 0.539). Measurements of the VCDR may have errors and differ significantly between modalities^30^. However, Cirrus HD-OCT can provide reliable and highly reproducible VCDR reference data to a neural network. We expect that these data may be more accurate than the VCDRs evaluated by human doctors.

This study had some limitations. We used the ONH as a target object. The ONH is not large in fundus photographs but is very prominent and contains rich characteristic features. However, modern state-of-the-art object-detection algorithms struggle to identify small objects^31^. Fundus photographs contain significantly smaller—but very important—objects (lesions), such as microaneurysms, hard exudates, soft exudates, and drusen. For detecting these extremely small objects, the performance may be degraded compared with our reported values. Moreover, fundus images contain amorphous objects, e.g., vitreous hemorrhages, epiretinal membranes, and geographic atrophies. These objects have various sizes (from very small to large) and unusual random aspect ratios. A regression-type objector such as YOLO V3 has difficulty finding these unusual-aspect ratio objects^32^. To evaluate the performance for detecting these amorphous objects, further study is necessary.

In conclusion, if only a CPU is used, a low input image resolution is recommended, and DenseNet had the best performance in this study. At a resolution of 224 × 224, its mean detection time was 394 ms, and its localization and classification accuracy was 80% and 0.065, respectively (mean IoU and MAE of the VCDR prediction, respectively). As the input image resolution increased, the overall performances (localization, classification, and diagnostic performance) all improved, and the difference among the architectures became practically insignificant. However, at a resolution of 832 × 832, the mean detection time without GPU assistance ranged from 4,395 to 11,365 ms. The detection was significantly accelerated when the GPU was used; even at the high resolution of 832 × 832, it was approximately 170 ms. Thus, researchers who desire optimal performance should increase the input resolution and consider using a GPU.

## Supplementary information

Supplementary Table S1.

## Data Availability

The datasets generated during and/or analysed during the current study are available from the corresponding author on reasonable request.
