# Supplementary material for: Automatic optic nerve head localization and cup-to-disc ratio detection using state-of-the-art deep-learning architectures
Source: Sci Rep. 2020 Mar 19;10:5025. doi: 10.1038/s41598-020-62022-x (PMC7081256; doi:10.1038/s41598-020-62022-x)
Supplement: Supplementary file 1 — Supplementary Table S1. [file 41598_2020_62022_MOESM1_ESM.pdf]

Automatic optic nerve head localization and cup-to-disc ratio detection using state-of-the-art deep-learning architectures

Keunheung Park, MD[1,3], Jinmi Kim, PhD[2], Jiwoong Lee, MD, PhD[1,3]

Author affiliations

[1]Department of Ophthalmology, Pusan National University College of Medicine, Busan, Korea

[2]Department of Biostatistics, Clinical Trial Center, Biomedical Research Institute, Pusan National University Hospital, Busan, Korea

[3]Biomedical Research Institute, Pusan National University Hospital, Busan, Korea

First Author: Keunheung Park, MD

Department of Ophthalmology, Pusan National University Hospital, 179 Gudeok-ro, Seo-gu, Busan 49241, South Korea

Tel: +82-10-5654-0115

E-mail: climyth@naver.com

Second Author: Jinmi Kim, PhD

Department of Biostatistics, Clinical Trial Center, Pusan National University Hospital, 179 Gudeok-ro, Seo-gu, Busan 49241, South Korea

Tel: +82-51-240-7738

E-mail: jmkim@pnuh.co.kr

Corresponding author: Jiwoong Lee, MD, PhD

Department of Ophthalmology, Pusan National University College of Medicine, 179 Gudeok-ro, Seo-gu, Busan 49241, Korea

Tel: 82-51-240-7326

Fax: 82-51-242-7341

E-mail: alertlee@naver.com

Supplementary Table S1. Training time and the number of iterations

Resolution	Statistics	YOLO V3	ResNet	DenseNet
224 × 224	Training time	22h 22m	17h 53m	13h 33m
	Iterations	34,500	31,800	27,900
416 × 416	Training time	25h 01m	23h 49m	16h 35m
	Iterations	27,100	31,900	30,000
832 × 832	Training time	33h 32m	43h 56m	17h 56m
	Iterations	21,000	23,700	32,400

Trained on Intel 8th generation central processing unit (CPU) (i5-8400, 2.81 GHz, 32 GB main memory) and NVIDIA Titan Xp (12 GB; Santa Clara, CA, USA).
